# Validation of a semi-quantitative Food Frequency Questionnaire for dietary assessment among adults in Northwest China

**DOI:** 10.3389/fnut.2026.1798833

**Published:** 2026-06-04

**Authors:** Jing Huang, Huinan Zhang, Xing Wang, Peiwen Shi, Huifeng Zhang, Qiang Zeng

**Affiliations:** 1 Health Management Institute, The Second Medical Center and National Clinical Research Center for Geriatric Diseases, Chinese PLA General Hospital, Beijing, China; 2 Department of Health Management, Tangdu Hospital, Fourth Military Medical University, Xi'an, Shaanxi, China

**Keywords:** dietary assessment, Food Frequency Questionnaire, Northwest China, reproducibility, validity

## Abstract

**Background:**

Accurately assessing habitual diet is essential for nutritional epidemiology. While food frequency questionnaires (FFQs) are a practical tool for large-scale studies, they require population-specific validation due to regional dietary variations. This study aimed to validate a culturally tailored semi-quantitative FFQ for an understudied population in Northwest China, utilizing a digital Chronic Disease Follow-up Platform (CDFP) to enhance data collection and participant engagement.

**Methods:**

A culturally specific 62-item FFQ was developed based on local dietary surveys. The FFQ was administered twice (FFQ1, FFQ2) 3 months apart to assess reproducibility. Relative validity was evaluated against the average of three non-consecutive 24-h food records (3d-FR). The CDFP was employed to manage the entire validation study workflow, including recruiting, sending automated short-message reminders for uploading photographs of food records, and scheduling follow-up telephone calls for the completion of two FFQ administrations (FFQ1, FFQ2). Nutrient intakes were calculated using the China Food Composition Database. Statistical analyses included correlations, intraclass correlation coefficients (ICCs), cross-classification into quartiles, weighted kappa statistics, and Bland-Altman plots.

**Results:**

The FFQ demonstrated good to excellent reproducibility, with a mean ICC of 0.71 between FFQ1 and FFQ2. Energy-adjusted correlation coefficients between the FFQ1 and FFQ2 ranged from 0.46 to 0.82 (mean: 0.70). For validity, cross-classification showed 77.1% of participants were assigned to the same or adjacent quartile on average, with gross misclassification into opposite quartiles averaging 5.2%. Bland-Altman analysis indicated acceptable agreement, though a systematic overestimation by the FFQ was observed for energy and most nutrients. However, we also observed modest to fair validity correlations (mean crude *r* = 0.31 and energy-adjusted *r* = 0.22), with several nutrients showing non-significant associations in crude analyses.

**Conclusions:**

The developed FFQ shows reasonable reproducibility and acceptable relative validity for ranking nutrient intakes rather than for estimating absolute intakes among adults in northwest China. The integration of the CDFP proved to be an effective strategy for avoiding loss to follow-ups and ensuring high-quality dietary data collection. Further validation studies in community-based settings with larger and more balanced samples are required.

## 1 Introduction

Dietary assessment is essential for understanding the relationship between nutrition and health outcomes, particularly in populations with specific metabolic conditions such as obesity (1). While traditional methods like food records and 24-h dietary recalls provide detailed dietary data, they impose significant participant and researcher burdens, making them impractical for large-scale studies (2, 3). In contrast, food frequency questionnaires (FFQs) offer a cost-effective and efficient means of assessing long-term dietary patterns, making them widely used in nutritional epidemiology (2, 4). However, FFQs are primarily designed for ranking participants by their relative nutrient intake levels rather than quantifying absolute dietary consumption, and their validity depends on cultural, regional, and population-specific dietary habits (5).

In China, dietary patterns vary significantly across regions due to differences in food availability, ethnic traditions, and socioeconomic factors. While several FFQs have been developed and validated for Chinese populations, most have either focused on specific purposes or used simple food items (6, 7). Northwest China, encompassing provinces such as Shaanxi, Gansu, Ningxia, and Xinjiang, exhibits unique dietary characteristics including high consumption of wheat-based foods, lamb, and dairy products. These distinct dietary patterns necessitate region-specific dietary assessment tools. Existing FFQs used in national nutrition surveys may not fully account for regional dietary variations in northwest China (8). Therefore, developing a culturally adapted and population-specific FFQ is crucial for reliable dietary assessment in this group.

The present study aimed to develop and validate a semi-quantitative FFQ specifically designed for adult residents in northwest China. This FFQ was evaluated for relative validity against multiple 24 h food records and test-retest reproducibility to ensure its accuracy in ranking nutrient and food group intake. A validated dietary assessment tool tailored to this region will enhance nutritional surveillance, facilitate research on diet-disease relationships, and support the development of targeted nutritional interventions for northwest China's diverse population.

## 2 Materials and methods

### 2.1 Subjects and design

This validation and reproducibility study of the FFQ was embedded within a randomized controlled trial (Registration: ChiCTR2400089342) examining the efficacy of a low glycemic index diet in weight maintenance among weight-loss individuals. Approved by the Institutional Ethics Committee of Tangdu Hospital, Fourth Military Medical University (Approval No.: KG202403-17), the study adhered to the ethical principles of the Declaration of Helsinki. We recruited 200 adults aged 18–60 years who was undergoing weight maintenance in the health management department of Tangdu Hospital from January to June 2025, excluding those with significant metabolic comorbidities. In the randomized controlled trial, the 200 participants were randomly assigned to either an intervention group or a blank control group evenly. For the present FFQ validation study, only the 100 participants from the blank control group—whose dietary habits remained free from external interference—were included. The follow-up period for the project was 3 months. During this time, the FFQ was administered twice (at baseline and at the final visit) to enable reproducibility analysis, and three non-consecutive 24-h dietary records were collected for validation purposes. During follow-up, 2 participants dropped out due to health reasons, resulting in a final sample of 98 participants. A flow diagram of participants was shown in Supplementary Figure S1. Trained interviewers conducted face-to-face interviews at the initial and following visits. In the initial interview, the information on demographic and lifestyle variables was obtained by using structured questionnaires. Written informed consent was obtained from all participants, with data anonymized to ensure confidentiality.

### 2.2 FFQ development

The development of the FFQ followed a comprehensive approach to ensure its cultural appropriateness and scientific validity for assessing dietary intake in northwest China. Drawing upon established methodologies as reviewed by Cade et al. (9, 10), we constructed a semi-quantitative FFQ comprising 62 food items categorized into 14 major food groups: staple foods (including regional wheat-based products, such as dumplings, noodles, steamed buns), red meats (with emphasis on processed and unprocessed red meat), poultry, offal, aquatic products, dairy, eggs, legumes, vegetables, fruits, nuts, beverages, snacks, and alcoholic drinks. Food items were selected based on their nutritional composition, regional consumption patterns from the China national nutrition survey (8), while portion sizes were estimated using both standard metrics (grams) and household measures (bowls, pieces) supplemented with a photographic atlas of common servings. The FFQ employed a 9-point frequency scale ranging from “almost never” to “5+ times per day” following previous methods (9, 10). Extra 12 questions about serving sizes, eating-out frequency, and taste preferences were additionally asked in the questionnaire.

The FFQ was designed to assess habitual dietary intake over the past 4 weeks. This reference period was selected to capture usual intake while minimizing recall burden. The FFQ was administered by trained interviewers, with an average completion time of 10–15 min. The participants were firstly asked to choose their average frequency of intake for each food or food group. If the frequency is not never, they were further asked to report the average numbers of standard portion size for each item, each time it was consumed. The trained interviewers read aloud the standard portion size of each food item for every question. Visual aids—including standardized food models and portion estimation photographs—were used to enhance accuracy during recall collection.

### 2.3 Dietary validation analysis

To validate the newly developed FFQ, we conducted a 3-day (including two weekdays and one weekend day) 24-h food record (3d-FR) during the 3-month interval of two administrations of the FFQ. A Chronic Disease Follow-up Platform (CDFP) detailed in supplementary methods was used in our study, which facilitated participant reminders and data collection. The CDFP improved participant response rates by sending automated text message reminders and placing follow-up telephone calls to schedule hospital visits. Participants were instructed to photograph all consumed foods and beverages–including meals, beverages, and snacks–and subsequently record detailed descriptions alongside uploading the photos as verification. The recording period covered two weekdays and 1 weekend day to capture habitual dietary patterns, with platform notifications prompting timely entries.

Prior to data collection, participants received standardized instructions about food documentation techniques, including how to describe food items (brands, portion sizes), and upload clear meal images. Emphasis was placed on maintaining typical eating habits during recording. Trained nutritionists reviewed all submissions for completeness, contacting participants for clarification when necessary, and coded entries using standardized protocols. The CDFP allowed participants to upload their meal images conveniently via their mobile phones, while the photographic evidence enhanced portion size accuracy and data reliability for subsequent comparison with FFQ results.

### 2.4 Nutrient intake analysis

Food consumption data from the 3d-FR were entered into Feihua Nutrition Software (http://www.yyjsq.cn, V2.8.0.5, Beijing, China), a dietary analysis program based on the China Food Composition Database, which includes over 6,900 food items and 90+ nutrients. To ensure data accuracy, all entries were cross-checked by two trained nutritionists. Daily nutrient intakes were calculated via the software by matching reported foods with their nutrient composition in the database, accounting for regional dishes through recipe decomposition when necessary. For mixed dishes and homemade meals, individual ingredients were entered separately.

Nutrient intakes derived from the FFQ were computed by multiplying reported food weights and their corresponding nutrient composition values together. For each food item, daily consumption quantities (g/day) were calculated by multiplying self-reported consumption frequencies (converted to daily values using standardized conversion factors, shown in Supplementary Table S1) by reported food weight each time consuming. These quantities were then multiplied by nutrient composition values obtained from the China Food Composition Tables (6th edition) to determine daily nutrient intakes per food item. Total daily intakes of energy, macronutrients, and micronutrients were subsequently calculated by aggregating nutrient values across all listed food items in the FFQ.

### 2.5 Statistical analysis

All statistical analyses were performed using STATA software (version 16.0, Stata Corp LP). The distribution of nutrient intake data was assessed using the Kolmogorov-Smirnov test. For normally distributed variables, data are presented as mean ± standard deviation (SD), and paired *t*-tests were employed to evaluate differences between the averaged FFQ and 3d FR, as well as between FFQ1 and FFQ2. For variables violating the normality assumption, descriptive statistics are reported as medians with interquartile ranges (25th−75th percentiles), and the Wilcoxon signed-rank test was used for comparative analyses between dietary assessment methods.

For comparative analyses between dietary assessment methods, we employed multiple complementary approaches to assess the reproducibility and validity. First, we calculated correlation coefficients (ρ) to evaluate the association between nutrient intakes from both methods, both for crude and energy-adjusted values [using the nutrient density method (2)]. For macronutrients (protein, carbohydrates, and fat), we calculated their proportional contribution to total energy intake (as percentages of total energy). All other nutrients were standardized per 1,000 kcal (4,186 kJ) of total energy intake. These energy-adjusted nutrient values were subsequently incorporated as primary variables in multiple logistic regression models, with total energy intake included as a covariate, following Willett's established methodological framework (2).

Secondly, we computed intraclass correlation coefficients (ICCs) with 95% confidence intervals between dietary assessment methods. Thirdly, we conducted quartile cross-classification analysis to evaluate the agreement in ranking participants' nutrient intakes. Participants were classified into quartiles based on intake distributions from both methods, with proportions classified into same, adjacent (±1 quartile), one-apart (±2 quartiles), and extreme opposite quartiles calculated. We further assessed agreement using weighted kappa statistics (κ), applying the following weights: 1 for exact agreement, 0.67 for adjacent quartiles, 0.33 for ±2 quartiles, and 0 for opposite quartiles. Kappa values were interpreted as: very good (>0.80), good (0.61–0.80), moderate (0.41–0.60), fair (0.21–0.40), and poor (< 0.20).

Bland-Altman analysis was further performed to evaluate agreement via plotting mean differences against average intakes with 95% limits of agreement (mean difference ± 1.96 SD) between dietary assessment methods. All analyses were conducted for both macro- and micro-nutrients, with particular attention to nutrients of key public health importance in our study population. All statistical tests were two-tailed with significance set at *p* < 0.05.

## 3 Results

### 3.1 General characteristics of study participants

Table 1 summarizes the general characteristics of the study participants stratified by gender, with women representing 79.6% (*n* = 78) and men 20.4% (*n* = 20). The mean age of participants was 35.3 ± 8.5 years, with balanced occupational distribution between manual (43.9%) and intellectual workers (45.9%). The majority of participants were of Han ethnicity (98.0%), married (76.5%), held a bachelor's degree or equivalent (68.4%), and reported moderate physical activity levels (58.2%). Most demographic characteristics were comparable between genders, though significant differences were observed in anthropometric measures, and smoking as well as alcohol use patterns. Women had mean height and weight measurements of 162.2 cm and 67.3 kg, respectively, while men measured 174.5 cm and 82.6 kg. Despite these differences, body mass index (BMI) showed no significant gender variation, with an overall mean of 25.9 kg/m^2^. Male participants demonstrated higher rates of current or former smoking and more frequent alcohol consumption compared to females. The Depression Anxiety Stress Scale scores were similar between genders, with a mean score of 32.5 across the cohort. Regarding sleep patterns, 70.4% of participants reported no sleep deficiency.

**Table 1:** The characteristics of the included participants.

Characteristics		Women (*N* = 78, 79.6%)	Men (*N* = 20, 20.4%)	*p*	Total (*N* = 98)
Age at baseline (years)		34.7 (8.4)	37.8 (8.7)	0.153	35.3 (8.5)
Ethnicity [*N* (%)]	Ethnic Han	77 (98.7)	19 (95.0)	0.124	96 (98.0)
	Others	1 (1.3)	1 (5.0)		2 (2.0)
Educational level [*N* (%)]	High school or lower	5 (6.4)	1 (5.0)	0.864	6 (6.1)
	Bachelor or equivalent	54 (69.2)	13 (65.0)		67 (68.4)
	Master degree or higher	19 (24.4)	6 (30.0)		25 (25.5)
Marital status [*N* (%)]	Married	57 (73.1)	18 (90.0)	0.089	75 (76.5)
	Single or widowed	20 (25.6)	1 (5.0)		21 (21.4)
	Separated or divorced	1 (1.3)	1 (5.0)		2 (2.1)
Occupation [*N* (%)]	No work	8 (10.3)	2 (10.0)	0.133	10 (10.2)
	Manual worker	38 (48.7)	5 (25.0)		43 (43.9)
	Intellectual worker	32 (41.0)	13 (65.0)		45 (45.9)
Income [*N* (%)]	More than ¥10,000	21 (26.9)	7 (35.0)	0.775	28 (28.6)
	¥5,000–10,000	35 (44.9)	8 (40.0)		43 (43.9)
	Less than ¥5,000	22 (28.2)	5 (25.0)		27 (27.5)
Height (cm)		162.2 (5.0)	174.5 (3.7)	< 0.001	164.7 (6.9)
Weight (Kg)		67.3 (10.7)	82.6 (9.2)	< 0.001	70.4 (12.1)
Body Mass Index (BMI; kg/m^2^)		25.6 (4.0)	27.1 (2.4)	0.113	25.9 (3.8)
Physical activity [*N* (%)]	Low level	11 (14.1)	3 (15.0)	0.694	14 (14.3)
	Moderate level	44 (56.4)	13 (65.0)		57 (58.2)
	High level	23 (29.5)	4 (20.0)		27 (27.5)
Depression Anxiety Stress Scale (score)		32.5 (7.6)	32.4 (6.7)	0.936	32.5 (7.4)
sleep insufficiency [*N* (%)]	No	52 (66.7)	17 (85.0)	0.109	69 (70.4)
	Yes	26 (33.3)	3 (15.0)		29 (29.6)
Smoking status [*N* (%)]	Never smoked	64 (82.0)	13 (65.0)	0.016	77 (78.6)
	Passive smoking	11 (14.1)	2 (10.0)		13 (13.3)
	Ex-smoker	1 (1.3)	3 (15.0)		4 (4.1)
	Current smoker	2 (2.6)	2 (10.0)		4 (4.1)
Alcohol drink [*N* (%)]	Almost never	51 (65.4)	9 (45.0)	0.001	60 (61.2)
	Occasionally	24 (30.8)	5 (25.0)		29 (29.6)
	Often	3 (3.8)	6 (30.0)		9 (9.2)

### 3.2 Reproducibility

Reproducibility analysis of nutrient intakes between the first and second FFQ administrations (FFQ1 and FFQ2) is shown in Table 2. The mean fat intake was comparable between administrations, with FFQ1 reporting 48.3 g (SD: 27.7 g) and FFQ2 reporting 50.8 g (SD: 22.3 g). Similar consistency was observed for some micronutrients: cholesterol (451.8 mg vs. 475.0 mg), vitamin A (298.4 μg vs. 288.8 μg), and iodine (167.9 μg vs. 163.0 μg). The crude Pearson correlation coefficients ranged from 0.66 (dietary fiber) to 0.89 (iodine), with an average correlation of 0.76. Following energy adjustment using the nutrient density method, correlations slightly decreased to a range of 0.46 (vitamin A) to 0.82 (iodine), with an average correlation of 0.70. Notably strong correlations (≥0.70) were observed for daily intakes of energy, protein, vitamin C, Niacin, iron, zinc, sodium, potassium, magnesium, phosphorus, and iodine between FFQ1 and FFQ2, indicating excellent test-retest reliability of the questionnaire over the 3-month interval.

**Table 2:** Reproducibility study: median nutrient intakes from FFQs, and difference test, Pearson correlation between FFQ1 and FFQ2.

Variables		FFQ1^a^	FFQ2^a^	Paired *t*-test *P*-values	Pearson correlation coefficients			
		Mean (SD)	Mean (SD)		Energy-unadjusted		Energy-adjusted	
Energy	(kcal/d)	1,347.7 (658.6)	1,496.7 (602.1)	0.002	0.73	< 0.001		
Protein	(g/d)	70.3 (34.3)	76.6 (31.3)	0.006	0.78	< 0.001	0.73	< 0.001
Carbohydrate	(g/d)	158.1 (80.8)	183.3 (80.5)	< 0.001	0.69	< 0.001	0.68	< 0.001
Fat	(g/d)	48.3 (27.7)	50.8 (22.3)	0.194	0.72	< 0.001	0.69	< 0.001
Fiber	(g/d)	8.9 (4.2)	12.0 (5.4)	< 0.001	0.66	< 0.001	0.48	< 0.001
Cholesterol	(mg/d)	451.8 (258.7)	475.0 (237.3)	0.197	0.75	< 0.001	0.54	< 0.001
Vitamin C	(mg/d)	113.2 (56.8)	177.0 (99.5)	< 0.001	0.71	< 0.001	0.70	< 0.001
Vitamin B2	(mg/d)	1.1 (0.5)	1.2 (0.5)	< 0.001	0.82	< 0.001	0.66	< 0.001
Niacin	(mg/d)	14 (7.3)	15.7 (6.5)	0.001	0.75	< 0.001	0.74	< 0.001
Vitamin A	(μg/d)	298.4 (211.1)	288.8 (166.1)	0.537	0.69	< 0.001	0.46	< 0.001
Calcium	(mg/d)	587.6 (290.2)	660.7 (298.7)	< 0.001	0.80	< 0.001	0.65	< 0.001
Iron	(mg/d)	20.0 (9.4)	23.6 (9.8)	< 0.001	0.81	< 0.001	0.80	< 0.001
Zinc	(mg/d)	10.5 (4.9)	11.7 (4.7)	< 0.001	0.79	< 0.001	0.79	< 0.001
Sodium	(mg/d)	1,099.2 (612.1)	1,190.5 (545.2)	0.040	0.73	< 0.001	0.76	< 0.001
Potassium	(mg/d)	2,116.5 (977.7)	2,538.5 (1,108.2)	< 0.001	0.79	< 0.001	0.71	< 0.001
Magnesium	(mg/d)	258.3 (118.3)	311.4 (130.9)	< 0.001	0.79	< 0.001	0.72	< 0.001
Phosphorus	(mg/d)	999.1 (470.8)	1,100.2 (459.2)	0.001	0.81	< 0.001	0.72	< 0.001
Copper	(mg/d)	2.3 (1.1)	2.8 (1.2)	< 0.001	0.76	< 0.001	0.64	< 0.001
Selenium	(μg/d)	46.5 (23.3)	49.3 (20.3)	0.076	0.76	< 0.001	0.62	< 0.001
Iodine	(μg/d)	167.9 (219.4)	163.0 (215.6)	0.631	0.89	< 0.001	0.82	< 0.001

^a^FFQ, Food Frequency Questionnaire conducted firstly at baseline (FFQ1) and at the final visit for the second time (FFQ2)

Reproducibility analysis demonstrated good to excellent agreement between the two FFQ administrations as shown in Table 3. Intraclass correlation coefficients (ICCs) for energy and nutrients ranged from 0.49 (95% CI: 0.33–0.64; dietary fiber) to 0.90 (95% CI: 0.86–0.93; iodine), with a mean ICC of 0.71 across all measured parameters. Macronutrients showed moderate to strong reproducibility, with ICCs of 0.76 (95% CI: 0.67–0.84) for protein, 0.65 (95% CI: 0.54–0.77) for carbohydrates, and 0.70 (95% CI: 0.60–0.80) for total fat.

**Table 3:** Intraclass correlation coefficients and percentage agreement in quartile distribution of nutrient intake between FFQ1^a^ and FFQ2^a^.

Variables	ICC^b^			Percentage agreement of quartiles				Weighted Kappa	
	Statistics	95% CI	*P*-value	Same	Adjacent	One apart	Opposite	Statistics	*P*-value
Energy	0.70	0.60, 0.80	< 0.001	59.8	32.0	7.2	1.0	0.61	< 0.001
Protein	0.76	0.67, 0.84	< 0.001	55.7	37.1	7.2	0.0	0.59	< 0.001
Carbohydrate	0.65	0.54, 0.77	< 0.001	59.8	27.8	11.3	1.0	0.57	< 0.001
Fat	0.70	0.60, 0.80	< 0.001	67.0	23.7	8.3	1.0	0.65	< 0.001
Fiber	0.49	0.33, 0.64	< 0.001	58.8	30.9	7.2	3.1	0.56	< 0.001
Cholesterol	0.75	0.66, 0.84	< 0.001	52.6	39.2	6.2	2.1	0.54	< 0.001
Vitamin C	0.40	0.23, 0.57	< 0.001	63.9	22.7	9.3	4.1	0.57	< 0.001
Vitamin B2	0.80	0.73, 0.87	< 0.001	71.1	18.6	10.3	0.0	0.69	< 0.001
Niacin	0.72	0.62, 0.82	< 0.001	53.6	37.1	9.3	0.0	0.55	< 0.001
Vitamin A	0.68	0.57, 0.78	< 0.001	55.7	37.1	7.2	0.0	0.59	< 0.001
Calcium	0.78	0.70, 0.86	< 0.001	61.9	28.8	9.3	0.0	0.62	< 0.001
Iron	0.75	0.66, 0.84	< 0.001	68.0	26.8	5.2	0.0	0.70	< 0.001
Zinc	0.77	0.68, 0.85	< 0.001	59.8	33.0	7.2	0.0	0.62	< 0.001
Sodium	0.71	0.62, 0.81	< 0.001	60.8	33.0	4.1	2.1	0.62	< 0.001
Potassium	0.72	0.62, 0.82	< 0.001	57.7	33.0	7.2	2.1	0.57	< 0.001
Magnesium	0.71	0.61, 0.81	< 0.001	65.0	27.8	6.2	1.0	0.65	< 0.001
Phosphorus	0.79	0.71, 0.86	< 0.001	70.1	25.8	3.1	1.0	0.72	< 0.001
Copper	0.68	0.57, 0.78	< 0.001	59.8	35.1	4.1	1.0	0.63	< 0.001
Selenium	0.75	0.66, 0.84	< 0.001	57.7	35.1	6.2	1.0	0.60	< 0.001
Iodine	0.90	0.86, 0.93	< 0.001	62.9	26.8	8.2	2.1	0.61	< 0.001

^a^FFQ, Food Frequency Questionnaire conducted firstly at baseline (FFQ1) and at the final visit for the second time (FFQ2); ^b^ICC, Intraclass correlation coefficients.

The reproducibility of nutrient intake estimates between FFQ1 and FFQ2 was further assessed through quartile classification analysis, as presented in Table 3. The proportion of participants classified into the same or adjacent quartiles ranged from 86.6% (vitamin C) to 95.9% (dietary phosphorus), with an average agreement of 91.7% across all nutrients. Exact quartile agreement was achieved for 61.1% of participants on average, with particularly high consistency observed for vitamin B2 (71.1%), dietary phosphorus (70.1%), and dietary fat (67.0%). Gross misclassification into opposite quartiles was infrequent, with a maximum of 4.1% (vitamin C) and an average misclassification rate of 1.1%. Weighted kappa statistics demonstrated moderate to good agreement for most nutrients, with values ranging from κ = 0.54 (cholesterol) to κ = 0.72 (dietary phosphorus).

**Figure 1:**
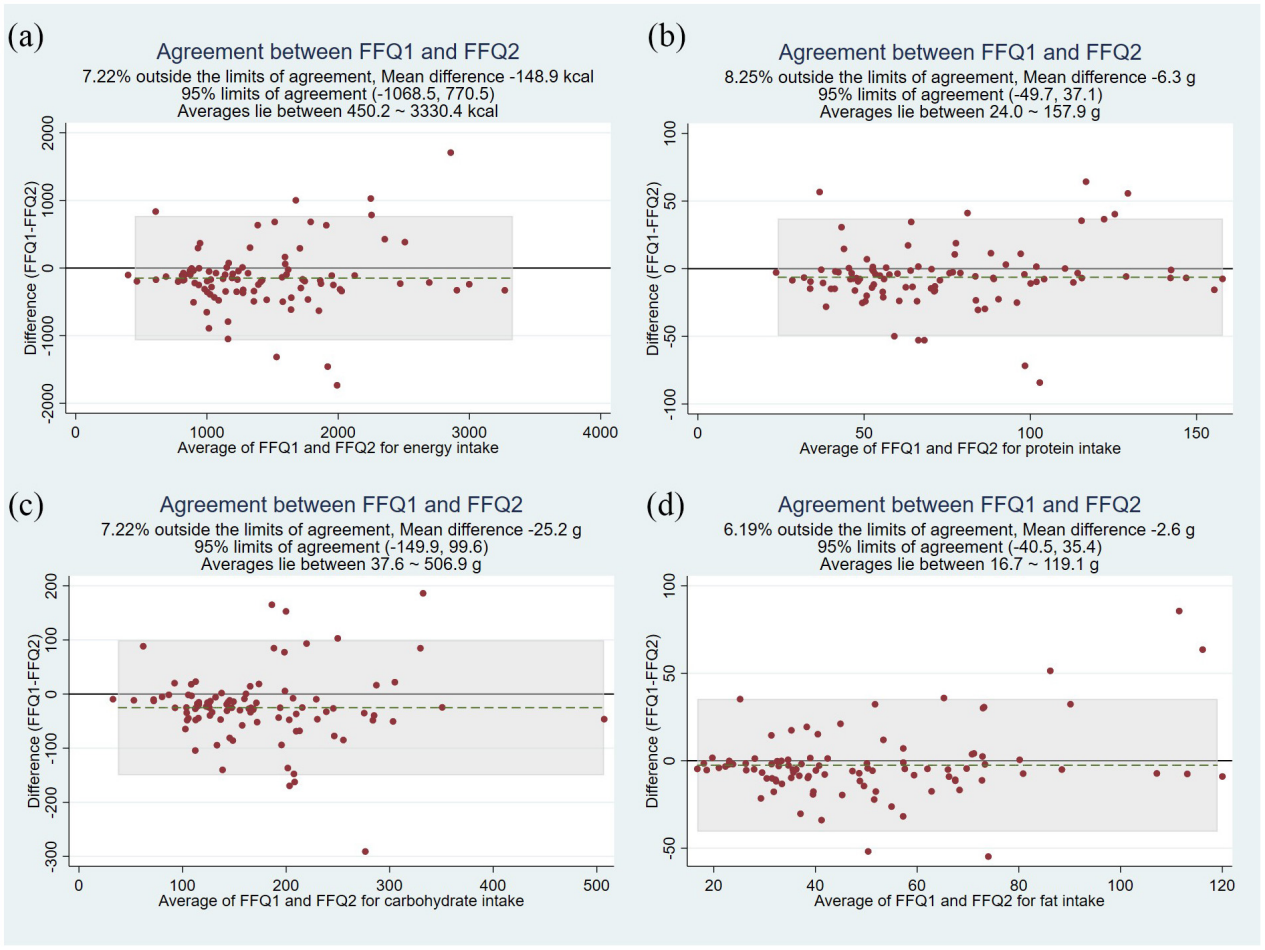
Bland–Altman plots showing agreement between FFQ1 and FFQ2 in estimating the in-takes of **(a)** energy, **(b)** protein, **(c)** carbohydrate and **(d)** fat.

The results of the Bland-Altman analysis for energy and macronutrients are presented in Figure 1, with corresponding results for micronutrients provided in Supplementary Figure S2. The analysis revealed systematic underestimation by FFQ1 compared to FFQ2 for most nutrients, with mean differences of −148.9 kcal for energy, −6.3 g for protein, −25.2 g for carbohydrate, and −2.6 g for total fat. These differences indicate that the second administration of the FFQ consistently yielded higher absolute intake estimates than the first. As shown in Figure 1 and Supplementary Figure S2, although a proportion of data points fell outside the 95% limits of agreement, the limits were generally acceptable: no nutrient exceeded 10% outliers, and on average, 93.1% of data points remained within the limits.

### 3.3 Validity

A comparison of nutrient intakes estimated by the averaged FFQ and the 3d-FR is presented in Table 4. The FFQ generally yielded higher estimates for most nutrients compared to the reference method, with mean energy intake measured at 1,428.7 ± 530.5 kcal vs. 1,244.5 ± 265.9 kcal for 3d-FR. Similar overestimation patterns were observed for macronutrients: protein (74.5 ± 29.2 g vs. 57.5 ± 15.2 g), fat (50.5 ± 22.5 g vs. 42.2 ± 13.8 g), but not for carbohydrates (169.4 ± 64.0 g vs. 159.4 ± 48.4 g). Correlation analysis revealed fair to moderate agreement between the two methods. Crude correlation coefficients ranged from 0.20 for fat to 0.41 for protein, with a mean correlation of 0.31 across all nutrients. Energy adjusted correlation coefficients ranged from 0.11 for Fiber to 0.44 for Zinc, with a mean correlation of 0.22 across all nutrients.

**Table 4:** Validation study: median nutrients intakes from FFQs, and difference test, Pearson correlation, between averaged FFQ^a^ and 3d-FR.

Variables		Averaged FFQ^a^	3d-FR^b^	Paired *t*-test *P*-values	Pearson correlation coefficients			
		Mean (SD)	Mean (SD)		Energy-unadjusted		Energy-adjusted	
Energy	(kcal/d)	1,428.7 (530.5)	1,244.5 (265.9)	0.002	0.35	0.002		
Protein	(g/d)	74.5 (29.2)	57.5 (15.2)	< 0.001	0.41	< 0.001	0.36	0.001
Carbohydrate	(g/d)	169.4 (64.0)	159.4 (48.4)	0.195	0.30	0.008	0.26	0.023
Fat	(g/d)	50.5 (22.5)	42.2 (13.8)	0.003	0.20	0.078	0.27	0.052
Fiber	(g/d)	10.7 (4.2)	12.6 (6.6)	0.015	0.25	0.024	0.11	0.331
Cholesterol	(mg/d)	468.8 (220.3)	433.8 (206.2)	0.233	0.27	0.018	0.17	0.144
Vitamin C	(mg/d)	150.2 (74.3)	115.1 (71.6)	0.001	0.23	0.045	0.14	0.209
Vitamin B2	(mg/d)	1.2 (0.4)	0.8 (0.3)	< 0.001	0.30	0.008	0.27	0.016
Niacin	(mg/d)	15.1 (6.5)	11.0 (3.6)	< 0.001	0.34	0.002	0.25	0.027
Vitamin A	(μg/d)	294.6 (167.6)	449.7 (333.0)	< 0.001	0.27	0.014	0.13	0.262
Calcium	(mg/d)	632.5 (251.7)	463.9 (152.7)	< 0.001	0.24	0.034	0.30	0.008
Iron	(mg/d)	22.1 (8.4)	15.6 (4.2)	< 0.001	0.45	< 0.001	0.26	0.020
Zinc	(mg/d)	11.2 (4.4)	7.8 (2.8)	< 0.001	0.40	< 0.001	0.44	< 0.001
Sodium	(mg/d)	1,167.9 (543.1)	1,093.3 (736.2)	0.399	0.28	0.011	0.13	0.270
Potassium	(mg/d)	2,372.3 (930.3)	1,685.9 (471.2)	< 0.001	0.32	0.004	0.19	0.094
Magnesium	(mg/d)	289.4 (109.8)	248.7 (75.8)	0.002	0.30	0.007	0.19	0.094
Phosphorus	(mg/d)	1,058.9 (402.0)	785.3 (189.0)	< 0.001	0.37	0.001	0.21	0.067
Copper	(mg/d)	2.6 (1.0)	1.8 (1.0)	< 0.001	0.38	< 0.001	0.16	0.166
Selenium	(μg/d)	48.2 (19.3)	37.5 (12.9)	< 0.001	0.32	0.005	0.18	0.110
Iodine	(μg/d)	166.2 (218.0)	87.6 (306.3)	0.039	0.23	0.039	0.18	0.117

^a^Averaged FFQ, average of FFQ1 and FFQ2, Food Frequency Questionnaire conducted firstly at baseline (FFQ1) and at the final visit for the second time (FFQ2); ^b^3d-FR: three-day 24-h food records.

The validity of the FFQ against the 3d-FR was evaluated through quartile classification analysis, with results detailed in Table 5. Classification consistency analysis revealed that 77.1% of participants on average were assigned to either identical or adjacent quartiles when comparing both methods, with values spanning from 67.1% for cholesterol to 83.5% for dietary copper. Energy demonstrated superior classification accuracy, showing the highest exact quartile agreement at 44.3%. The degree of opposite misclassification remained limited, averaging 5.2% across all nutrients and not exceeding 8.9% for any individual nutrient. Agreement metrics further substantiated these findings, with weighted kappa values ranging from κ = 0.20 (vitamin B2) to κ = 0.45 (energy), indicating fair to moderate validity overall.

**Table 5:** Intraclass correlation coefficients and percentage agreement in quartile distribution of nutrient intake between averaged FFQ^a^ and 3d-FR.

Variables	ICC^b^			Percentage agreement of quartiles				Weighted Kappa	
	Statistics	95% CI	*P*-value	Same	Adjacent	One apart	Opposite	Statistics	*P*-value
Energy	0.29	0.04, 0.54	0.022	44.3	34.2	15.2	6.3	0.45	< 0.001
Protein	0.21	−0.03, 0.45	0.087	32.9	43.0	21.5	2.5	0.35	0.001
Carbohydrate	0.36	0.13, 0.60	0.002	34.2	38.0	24.0	3.8	0.32	0.003
Fat	0.14	−0.10, 0.37	0.250	34.2	41.8	15.2	8.8	0.31	0.005
Fiber	0.20	−0.01, 0.41	0.063	38.0	39.2	17.7	5.1	0.37	< 0.001
Cholesterol	0.28	0.06, 0.50	0.011	38.0	29.1	27.8	5.1	0.35	0.007
Vitamin C	0.16	−0.05, 0.37	0.135	35.4	45.6	12.7	6.3	0.27	< 0.001
Vitamin B2	0.10	−0.15, 0.35	0.439	22.8	54.4	13.9	8.9	0.20	0.041
Niacin	0.12	−0.10, 0.34	0.287	34.2	41.8	19.0	5.0	0.34	0.002
Vitamin A	0.12	−0.09, 0.33	0.248	35.4	40.5	16.5	7.6	0.23	0.002
Calcium	0.06	−0.21, 0.32	0.675	35.4	39.3	19.0	6.3	0.33	0.003
Iron	0.12	−0.13, 0.38	0.345	32.9	44.3	20.3	2.5	0.35	0.001
Zinc	0.12	−0.11, 0.36	0.302	39.2	41.8	13.9	5.1	0.42	< 0.001
Sodium	0.26	0.07, 0.46	0.009	38.0	40.5	17.7	3.8	0.40	< 0.001
Potassium	0.04	−0.21, 0.29	0.731	30.4	51.9	12.7	5.0	0.35	0.001
Magnesium	0.26	0.03, 0.49	0.026	36.7	39.2	20.3	3.8	0.37	< 0.001
Phosphorus	0.11	−0.16, 0.37	0.437	40.5	39.2	16.5	3.8	0.43	< 0.001
Copper	0.21	−0.01, 0.43	0.058	39.2	44.3	11.4	5.1	0.43	< 0.001
Selenium	0.19	−0.04, 0.43	0.108	35.4	43.0	16.5	5.1	0.37	< 0.001
Iodine	0.19	−0.01, 0.39	0.068	36.7	38.0	20.2	5.1	0.25	0.001

^a^Averaged FFQ, average of FFQ1 and FFQ2, Food Frequency Questionnaire conducted firstly at baseline (FFQ1) and at the final visit for the second time (FFQ2); ^b^ICC, Intraclass correlation coefficients.

**Figure 2:**
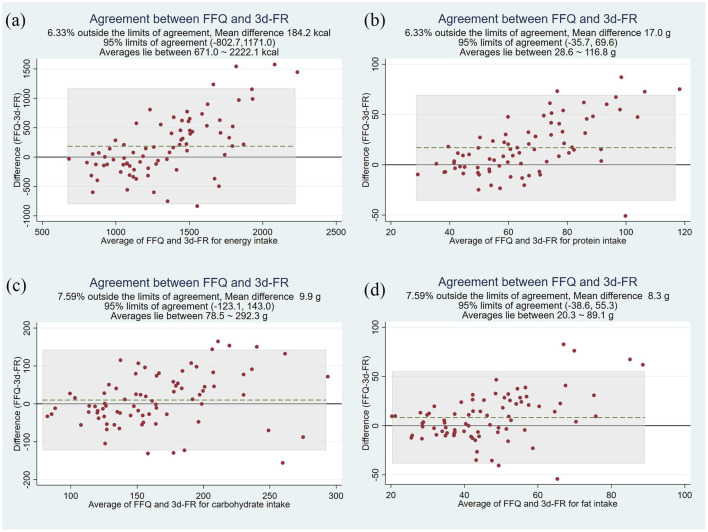
Bland–Altman plot showing agreement between the averaged FFQ and the 3d-FR in estimating the intakes of **(a)** energy, **(b)** protein, **(c)** carbohydrate and **(d)** fat.

The agreement between the averaged FFQ and 3d-FR was further examined through Bland-Altman analysis. As shown in Figure 2, systematic overestimation by the FFQ was observed for most nutrients, with mean differences of +184.2 kcal for total energy intake, +17.0 g for protein, +9.9 g for carbohydrate, and +8.3 g for total fat. The proportion of data points falling outside the 95% limits of agreement averaged 5.4% across all nutrients, remaining below 10% for all nutrients analyzed, suggesting generally acceptable agreement between the two dietary assessment methods (Figure 2 and Supplementary Figure S3).

## 4 Discussion

This study developed and validated a semi-quantitative FFQ specifically designed to assess habitual dietary intake among adults in northwest China. Our findings demonstrate that the developed FFQ possesses reasonable reproducibility and acceptable relative validity for ranking nutrient intakes rather than for estimating absolute intakes among adults in northwest China. To enhance generalizability and reliability, further validation studies are needed in community-based settings with larger sample sizes and more balanced participant characteristics.

The reproducibility analysis between FFQ1 and FFQ2 indicated good test-retest reliability, with intraclass correlation coefficients (ICCs) averaging 0.71 and high proportions of participants correctly classified into the same or adjacent quartiles. This suggests that the questionnaire provides a stable estimate of habitual intake over a 3-month period. In terms of validity, energy-adjusted correlation coefficients between the averaged FFQ and the 3d-FR ranged from 0.11 to 0.44, with minerals, particularly zinc and calcium, showing better agreement. These correlation values are comparable to those reported in other regional validation studies within China. For instance, a validation study of a FFQ in Southern China found correlations from 0.12 to 0.54 for most nutrients (11), while validation of a FFQ among 131 participants in Northeast China reported energy-adjusted Spearman correlation coefficients ranging from 0.26 to 0.55 (12). Although the Shanghai Diet and Health Study reported higher correlations for its FFQ validation, ranging from 0.33 to 0.77, the data were collected between 2012 and 2013, a period when dietary diversity in China was still relatively limited, which may have reduced variation between dietary assessment methods and contributed to the stronger agreement observed (13).

In assessing the agreement between the developed FFQ and the 3d-FR, we acknowledge that the observed validity correlations (mean crude *r* = 0.31 and energy-adjusted *r* = 0.22) are modest to fair, with several nutrients showing non-significant associations in crude analyses. Whereas correlation coefficients alone offer a limited perspective and multiple validation methods always are used (14). Cross-classification analysis provides a more clinically relevant measure of an instrument's ability to correctly rank individuals, which is crucial for investigating diet-disease relationships (15). In the present study, a mean of 77.1% of participants were classified into the same or adjacent quartiles across all nutrients, while severe misclassification into opposite quartiles averaged only 5.2%. These findings indicate that the FFQ is better suited for ranking individuals according to their nutrient intake rather than for estimating absolute intake values. This distinction is critical, as FFQs are designed primarily for epidemiological studies investigating diet-disease associations, where relative ranking of nutrient exposure is essential for examining relationships between diet and health outcomes (16). This level of agreement is comparable to, and in some cases surpasses, that reported in other regional validation studies within China. For instance, a study in Shanghai reported correct adjacent classification rates of 65–85% for major nutrients (17), while research among pregnant women in central China found similar ranges of 51.2–80.5% (18). Gross misclassification into the opposite quartile averaged 5.2% with the maximum no more than 10% in our study, reflecting a good performance in agreement between the developed FFQ and the 3d-FR. However, researchers using this instrument should avoid interpreting FFQ-derived estimates as absolute measures of intake and should employ energy adjustment to account for measurement error.

The Bland-Altman analysis provided valuable insight into the nature of the measurement error (19). The observed tendency of the FFQ to overestimate absolute intake, especially for energy and fat, is a common characteristic of FFQs and has been widely documented (2, 20). Several methodological factors may explain the systematic overestimation observed for most nutrients. First, the FFQ's portion size estimation method, which relies on standard portion photographs and household measures, may encourage participants to select ‘typical' portions that are larger than their actual consumption, particularly for commonly consumed foods. Second, the presence of certain food items in FFQs, such as vegetables, which can be consumed in mixed dishes, may lead to double-counting of similar foods consumed across different dishes (9). Third, systematic reporting bias and the cognitive challenges associated with recalling long-term consumption may also contribute to overestimation (20). Conversely, the underestimation of vitamin A observed in the Bland-Altman analysis is another characteristic previously documented for FFQs (10). This may reflect the FFQ's limited representation of vitamin A-rich foods, such as dark leafy vegetables and organ meats, which are less frequently consumed in northwest China. Nevertheless, the Bland-Altman analysis in our study demonstrated generally acceptable limits of agreement, with only a small proportion of data points falling outside these limits.

The energy adjustment of nutrient intakes proved to be a critical step, improving correlation coefficients and classification agreement for most parameters (21). This aligns with established methodological recommendations for reducing extraneous variation in dietary data (22). The use of weighted kappa statistics further strengthened our assessment by accounting for chance agreement in the cross-classification, with results (κ = 0.20–0.45) falling within the fair-to-moderate range typical for FFQ validation studies. For example, a validation study of an FFQ in New Zealand adults reported similar kappa values ranging from 0.12 to 0.47 for energy-adjusted nutrients (23). The relatively lower agreement observed for vitamin A and vitamin B2 is not uncommon, as the intake of these micronutrients is often influenced by supplement use and seasonal variation in food consumption, factors that are challenging to capture accurately with any dietary assessment tool (23, 24).

A key strength of our FFQ lies in its regional specificity. Unlike nationally developed tools, our questionnaire was constructed using a food list derived from local dietary surveys and market-basket analyses, ensuring the inclusion of staple foods and dishes characteristic of northwest China, such as wheat-based noodles (Lamian), baked breads (naan), and lamb-based dishes. This contrasts with FFQs developed for coastal or southern Chinese populations, which emphasize rice, seafood, and different vegetable varieties (11, 25). This cultural and dietary adaptation likely contributed to the relatively good performance observed for most nutrients. The structured use of a photographic atlas for portion size estimation, adapted from the methods of other digital photographic food atlas (26, 27), further enhanced the accuracy of intake quantification compared to instruments relying solely on text-based descriptions.

Another strength of our study is the usage of a digital administration platform (CDFP), which facilitated standardized data collection and reduced clerical errors. The CDFP was employed to manage the entire validation study workflow, including recruiting, sending automated short-message reminders for uploading photographs of food records, and scheduling follow-up telephone calls for the completion of two FFQ administrations (FFQ1, FFQ2). The usage of digital administration platform provided timely notification of follow-ups for participants to avoid unnecessary dropouts (28). Other strengths in our study should be noted. For instance, the validation was conducted against multiple (3-day) non-consecutive food records, a robust reference method that captures intra-individual variation (29). Moreover, the comprehensive statistical analyses, including correlation, intraclass correlation coefficients, cross-classification, weighted kappa analysis, and Bland-Altman agreement analyses, provided a multi-faceted assessment of the FFQ's performance. Although this FFQ was developed specifically for adults in Northwest China, the methodological framework employed has broader relevance for researchers in other global settings where region-specific dietary assessment tools are needed. The key strengths of our FFQ validation study can be readily replicated and adapted by investigators worldwide for diverse validation studies.

However, several limitations must be acknowledged. The use of 3-day food records as the reference standard, while practical, is itself subject to measurement error and may not perfectly represent true long-term intake (16). Biomarkers of intake, such as urinary nitrogen or doubly labeled water, were not employed, which are considered a superior validation standard for energy and certain nutrients (30). Additionally, as with all FFQs, the instrument remains susceptible to recall bias and social desirability bias. Furthermore, other limitations regarding generalizability need consideration. First, participants were recruited from the blank control group of a weight-management program, where individuals likely possess greater awareness of their dietary intake, potentially introducing reporting bias. Second, although recruitment from a major tertiary hospital setting in Northwest China enhanced the representativeness of the sample, the single-center design remains a limitation. Third, the predominance of female participants (79.6%) and the modest final sample size (*n* = 98) may have limited the generalizability of this study. Consequently, our findings may not fully represent the broader, more diverse adult population of northwest China, and future validation studies in community-based settings with larger and more balanced samples are required.

Future research should aim to validate this FFQ against objective biomarkers in a subsample to further confirm its accuracy. Additionally, testing its applicability in other sub-populations within the region, such as different age groups or ethnic minorities, is warranted. The integration of this culturally adapted FFQ into ongoing and future cohort studies will enable more precise investigations into the associations between the unique dietary patterns of northwest China and various health outcomes, such as cardiovascular diseases and chronic respiratory diseases, which exhibit distinct epidemiological patterns in this region (31, 32).

In conclusion, the Northwest China FFQ developed in this study demonstrated good measurement properties for ranking individuals according to their habitual intake of macronutrients and major micronutrients. Its region-specific design makes it a valuable tool for researchers seeking to elucidate diet-disease relationships in this understudied population, thereby contributing to the development of targeted, culturally appropriate nutritional public health strategies.

## Data Availability

The raw data supporting the conclusions of this article will be made available by the authors, without undue reservation.
