# Supplementary material for: Validation of a semi-quantitative Food Frequency Questionnaire for dietary assessment among adults in Northwest China
Source: Front Nutr. 2026 Jun 4;13:1798833. doi: 10.3389/fnut.2026.1798833 (PMC13266591; doi:10.3389/fnut.2026.1798833)
Supplement: Supplementary file 1 [file Table_1.docx]

Supplementary Material

# Supplementary Methods

The digital Chronic Disease Follow-up Platform (CDFP) used in this study is the **Tangdu Intelligent Chronic Disease Management Platform**, developed in 2022-2025. It is a comprehensive research support system designed for prospective cohort study management. The platform enables continuous participant tracking, automated follow-up reminders, and systematic data quality control.

The platform consists of four components: a PC-based management console, a researcher application, a participant application, and a WeChat mini-program. Key research-oriented functional modules include:

1. **Participant and cohort management:** supporting random grouping, stratified allocation, and intelligent management of study cohorts;
2. **Electronic Data Capture System:** enabling digital data collection, response tracking, and validation to ensure data integrity and safety.
3. **Multi-source data integration module:** interfacing with hospital electronic medical records and examination databases to combine clinical and research data;
4. **Automated follow-up module:** utilizing SMS and voice call reminders to standardize follow-up procedures, improve response rates, and reduce manual workload.

The CDFP addresses common challenges in follow-up studies, including low rates of follow-ups, data missing, and heavy reliance on manual operations. It provides researchers with a traceable, high-quality, and standardized tool for longitudinal data collection and management, making it well-suited for nutritional epidemiology and other cohort-based research.

In our study, all participants were asked to enrolled into the CDFP at the baseline visit. The platform then triggered automated SMS and voice call reminders for at least three non-consecutive 24-hour dietary records, during which participants uploaded food photographs via the mobile application. Researchers remotely monitored submissions and issued automated prompts for incomplete records. At the three-month follow-up, the platform reminded participants to visit the researcher center again for investigation. All data were integrated into a centralized database with automated quality control checks, ensuring standardized and traceable longitudinal data collection.

# Supplementary Figures and Tables

## Supplementary Tables

Supplementary Table S1 Transformation from intake frequencies to daily times using standardized conversion factors.

| Frequency of intake | Standardized conversion factors (formula) |
| --- | --- |
| Never or almost never | 0 |
| Less than once a month | 0.02 (0.5/30) |
| 1-3 per month | 0.07 (2.0/30) |
| Once a week | 0.14 (1.0/7) |
| 2-4 per week | 0.40 (3.0/7) |
| 5-6 per week | 0.80 (5.5/7) |
| Once per day | 1.00 (1.0/1) |
| 2-4 per day | 3.00 (3.0/1) |
| 5+ per day | 5.00 (5.0/1) |

## Supplementary Figures


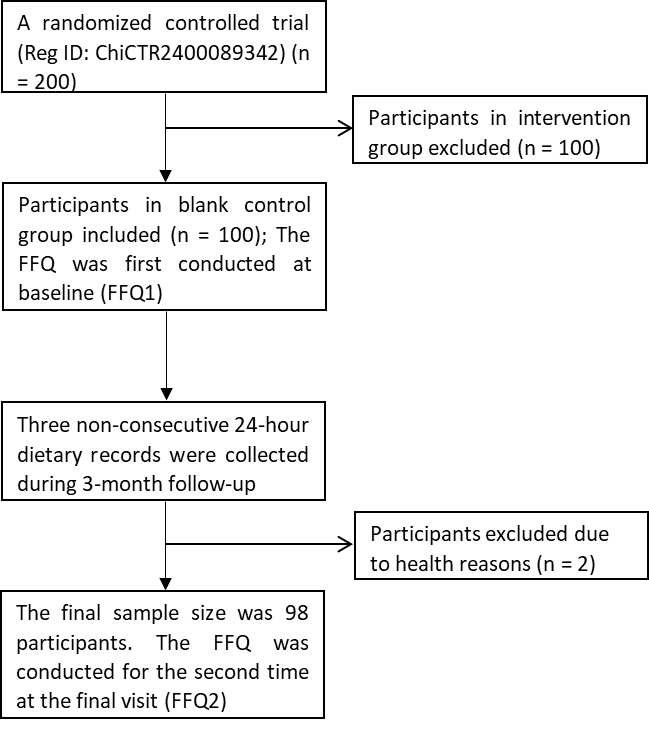


**Supplementary Figure S1.** Schematic overview of the study timeline, including dietary data collection points and participant flow throughout the validation study


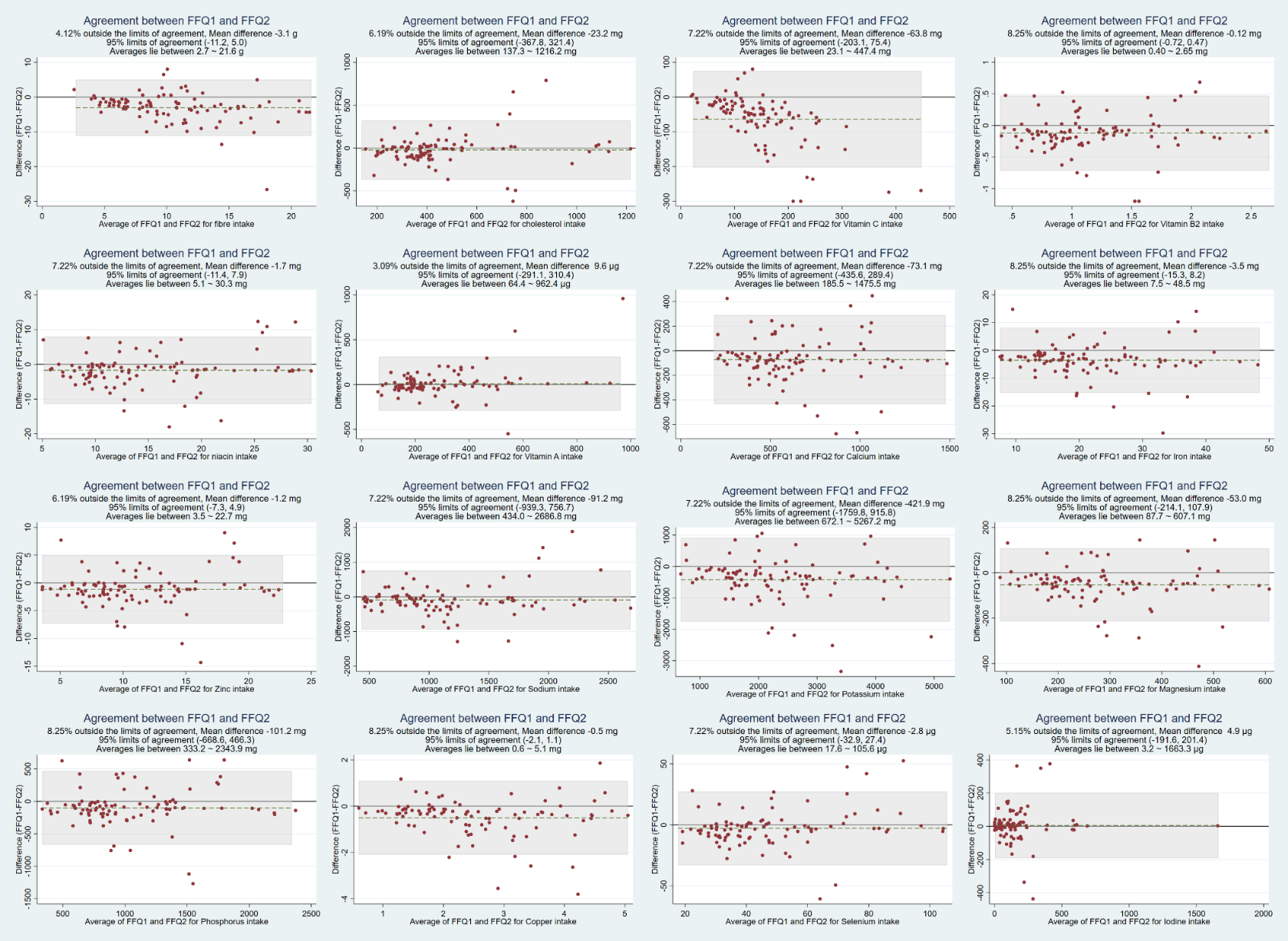


**Supplementary Figure S2.** Bland–Altman plots showing agreement between FFQ1 and FFQ2 in estimating the intakes of micronutrients.


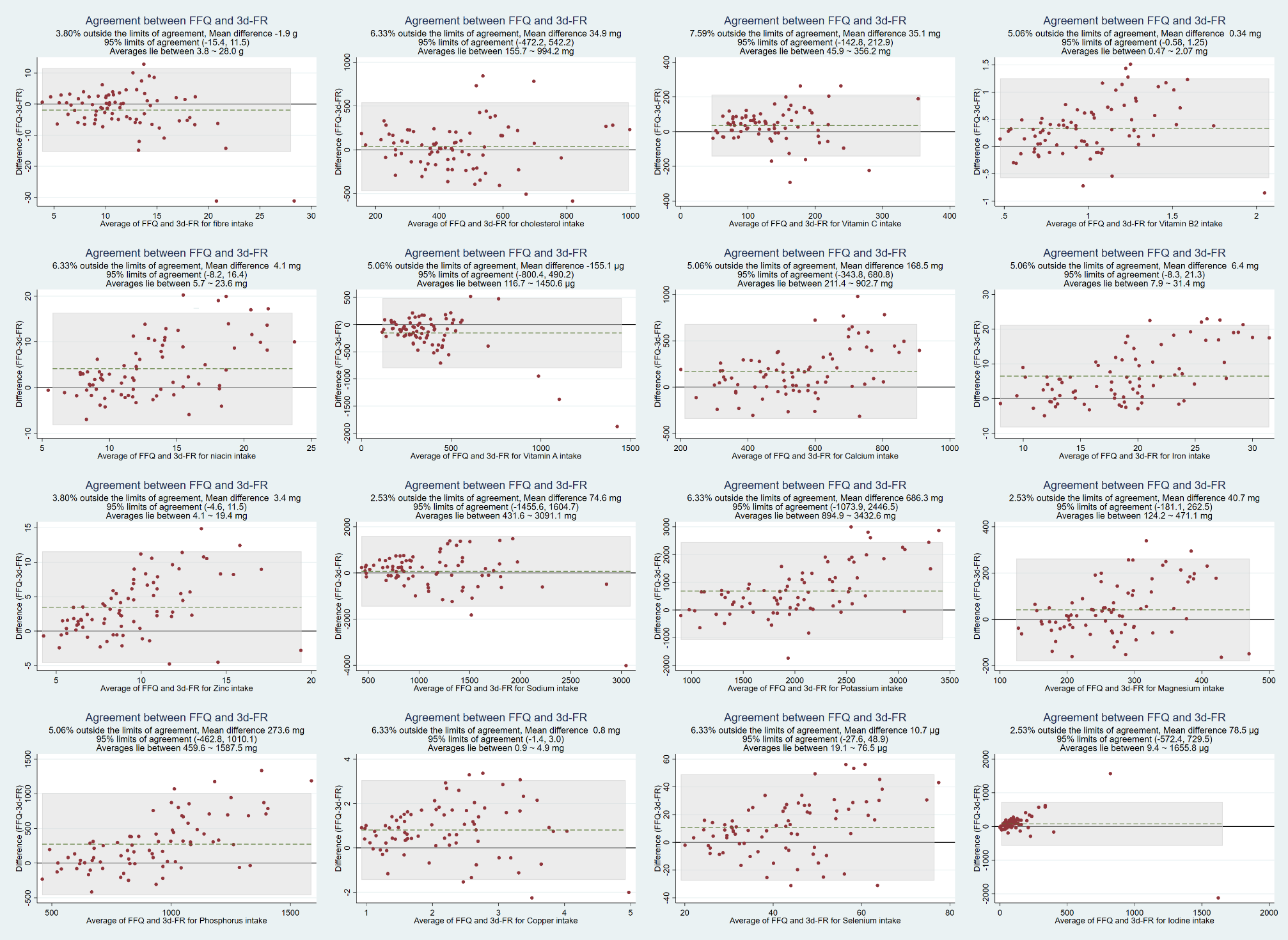


**Supplementary Figure S3.** Bland–Altman plot showing agreement between the averaged FFQ (the average of FFQ1 and FFQ2) and the 3d-FR in estimating the intakes of micronutrients. (FFQs - food frequency questionnaires, 3d-FR - three-day 24-hour food records)
